# Leaf Age-Dependent Photoprotective and Antioxidative Response Mechanisms to Paraquat-Induced Oxidative Stress in *Arabidopsis thaliana*

**DOI:** 10.3390/ijms160613989

**Published:** 2015-06-18

**Authors:** Julietta Moustaka, Georgia Tanou, Ioannis-Dimosthenis Adamakis, Eleftherios P. Eleftheriou, Michael Moustakas

**Affiliations:** 1 Department of Botany, School of Biology, Aristotle University of Thessaloniki, University Campus, 54124 Thessaloniki, Greece; E-Mails: grad738@edu.biology.uoc.gr (J.M.); iadamaki@bio.auth.gr (I.-D.A.); eelefth@bio.auth.gr (E.P.E.); 2 Faculty of Agriculture, Forestry and Natural Environment, School of Agriculture, Aristotle University of Thessaloniki, 54124 Thessaloniki, Greece; E-Mail: gtanou@agro.auth.gr

**Keywords:** antioxidant enzymes, chlorophyll fluorescence, differential response, electron transport, herbicides, hydrogen peroxide, non-photochemical quenching, reactive oxygen species

## Abstract

Exposure of *Arabidopsis thaliana* young and mature leaves to the herbicide paraquat (Pq) resulted in a localized increase of hydrogen peroxide (H_2_O_2_) in the leaf veins and the neighboring mesophyll cells, but this increase was not similar in the two leaf types. Increased H_2_O_2_ production was concomitant with closed reaction centers (*q*_P_). Thirty min after Pq exposure despite the induction of the photoprotective mechanism of non-photochemical quenching (NPQ) in mature leaves, H_2_O_2_ production was lower in young leaves mainly due to the higher increase activity of ascorbate peroxidase (APX). Later, 60 min after Pq exposure, the total antioxidant capacity of young leaves was not sufficient to scavenge the excess reactive oxygen species (ROS) that were formed, and thus, a higher H_2_O_2_ accumulation in young leaves occurred. The energy allocation of absorbed light in photosystem II (PSII) suggests the existence of a differential photoprotective regulatory mechanism in the two leaf types to the time-course Pq exposure accompanied by differential antioxidant protection mechanisms. It is concluded that tolerance to Pq-induced oxidative stress is related to the redox state of quinone A (Q_A_).

## 1. Introduction

In plants, most environmental stresses provoke oxidative damage due to excessive accumulation of reactive oxygen species (ROS), such as superoxide anions (O_2_·^−^), hydrogen peroxide (H_2_O_2_), hydroxyl radical (OH·), and singlet oxygen (^1^O_2_) [1]. Chloroplasts are the main targets of ROS-linked damage during various environmental stresses that may lead to leaf senescence and to cell death [2,3,4]. Leaf senescence is a genetically programmed decline in various cellular processes including photosynthesis and it is governed by leaf age, being induced or enhanced by environmental stresses [4]. Excess intracellular ROS reacts with a large variety of biomolecules and causes irreversible cellular damage leading to programmed cell death [5]. Photo-oxidation processes should be mitigated to prevent formation of ROS, and thus prevent leaf senescence [6]. However, in plants under oxidative stress conditions, over-accumulation of ROS is followed by the induction of different genes involved in detoxification stress response and defense [7].

In the Mehler reaction, reduction of O_2_ results in production of O_2_·^−^ that via a disproportionation reaction catalyzed by superoxide dismutase (SOD) is converted to H_2_O_2_ [8,9]. H_2_O_2_ produced by disproportionation of O_2_·^−^, causes photooxidative stress and destroys rapidly the chloroplast membranes [10,11]. Ascorbate peroxidase (APX) in chloroplasts catalyzes the conversion of H_2_O_2_ to water using ascorbate as electron donor [1]. In chloroplasts, it is possible to generate O_2_·^−^ by exposure of leaves to paraquat (Pq) [8,12]. Pq (methyl viologen) is a widely used non-selective herbicide for a variety of agricultural crops and has been classified among the most toxic and hazardous herbicides, being toxic to plants, animals, and humans [12,13,14]. Since only a small amount of applied herbicides actually reaches the target plants their release to soil and water leads to chemical pollution, affecting growth and fitness of non-target organisms [12]. Pq is a redox-active molecule that is taken up very quickly by leaves, undergoing univalent reduction by electrons donated from photosystem I (PSI) and subsequently transferring their electrons to oxygen, forming O_2_·^−^ and regenerating oxidized Pq, that may be engaged in successive rounds of redox cycling [11,15]. The extent to which the antioxidant enzymes APX and SOD can counteract the overproduction of ROS may determine whether or not plant cells survive [5,7,12,15].

*Arabidopsis* plants are protected from Pq exposure by increasing non-photochemical quenching (NPQ) that dissipates light energy and decreases the efficiency of photochemical reactions of photosynthesis by reducing electron transport rate (ETR) [12]. Moreover, differential response of young and mature leaves to herbicides has been reported [16,17]. In addition, young leaves of *A. thaliana* acclimate better to the onset of water deficit by dissipating the excess excitation energy by NPQ [18]. Excess energy dissipation associated with energy quenching of chlorophyll fluorescence (qE) functions as an efficient photoprotection mechanism in photosystem II (PSII) [19,20]. Yet, acclimation of young but not of mature leaves to moderate water deficit-induced oxidative stress was achieved by a reduced excitation pressure and a better balance between light capture and photochemical energy use, which contributed to their photoprotection [21].

During their lifetime leaves need to adapt to a changing environment that results in ROS production and oxidative stress. Photo-oxidative stress leading to photo-oxidation processes occurs when ROS production is not counterbalanced by antioxidant defenses [6]. Thus by estimating if ROS production is controlled by the antioxidant system, the photoprotective mechanisms can be elucidated. A differential ROS formation or scavenging in young and mature leaves can reveal the physiological mechanisms to abiotic stress acclimation and leaf senescence.

One of the most important photoprotective mechanisms is NPQ of excitation energy [19,22]. Chlorophyll fluorescence, and in particular pulse amplitude-modulated (PAM) fluorometry has become by far the dominant technique to measure NPQ and has proven to be a useful, noninvasive tool for the estimation of inhibition or damage in the PSII electron transfer process [22,23,24,25,26,27]. In the present study, we aimed to estimate the differential response of young and mature leaves to Pq-induced oxidative stress and to try to gain an insight into the mechanisms that play a role in the antioxidant and photoprotective process. The overall hypothesis was that *Arabidopsis thaliana* young leaves would reveal a better photoprotection with reduced excitation pressure and a better antioxidant mechanism resulting in a better ROS homeostasis than mature leaves. *A. thaliana* was chosen for the study of Pq-induced oxidative stress since it has been widely used as a model system in understanding the physiological mechanisms of higher plants [12].

## 2. Results

### 2.1. Non-Photochemical Quenching Increased and Electron Transport Decreased in Response to Paraquat Exposure

**Figure 1:**
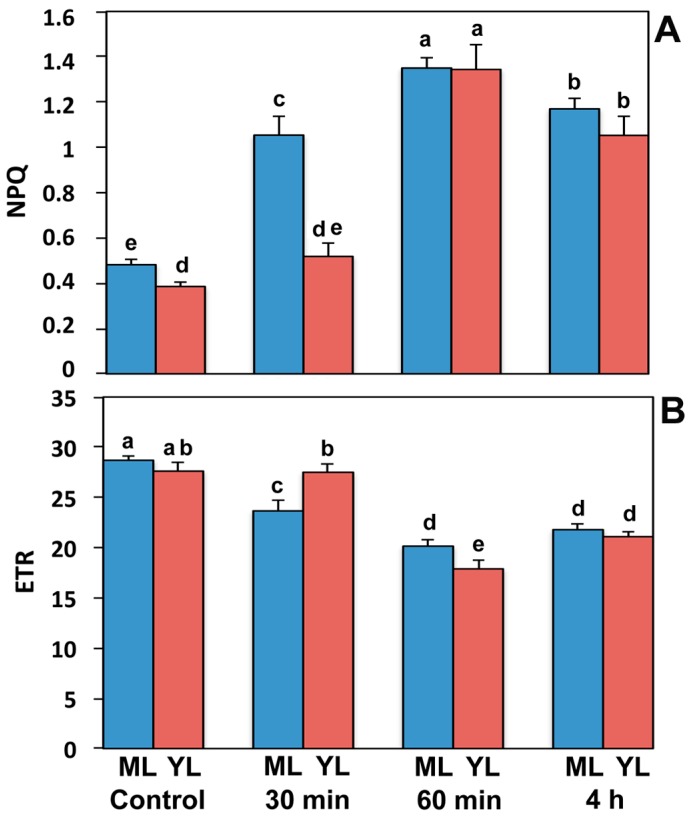
Changes of (**A**) non-photochemical quenching (NPQ); and (**B**) electron transport rate (ETR), of control and after 30 min, 60 min, and 4 h Pq exposure of *A. thaliana* mature (ML) and young leaves (YL). Blue columns: mature leaves; red columns: young leaves. Data shown are means ± SE indicated by bars (*n* = 6). Columns with different lowercase letters are significantly different (*p* < 0.05).

The non-photochemical quenching (NPQ) that reflects heat dissipation of excitation energy in the antenna system and serves as a photoprotective mechanism, increased in both leaf types due to Pq exposure (Figure 1A). In young leaves 30 min after Pq treatment, NPQ shows only a tendency to increase (ns, non significant increase), while mature leaves increased by 118% (*p* = 0.0002) compared to control. Sixty min after Pq treatment, NPQ increased by 180% (*p* < 0.0001) and 249% (*p* = 0.0005), in mature and young leaves respectively, compared to their controls (Figure 1A). Four hours after Pq treatment, NPQ decreased by 13% (*p* < 0.05) and 22% (*p* < 0.005) in mature and young leaves respectively, compared to 60 min after Pq treatment, thus resulting to an increase of 143% (*p* < 0.0001) and 173% (*p* = 0.0001) in mature and young leaves respectively, compared to their controls (Figure 1A).

The electron transport rate (ETR) in both leaf types reduced after exposure to Pq (Figure 1B). In young leaves, 30 min after Pq exposure, ETR had a non-significant decreasing tendency compared to control, while in mature leaves it decreased by 18% (*p* < 0.05). Sixty min after Pq exposure the ETR decreased by 15% (*p* < 0.0001) and 35% (*p* < 0.0001), in mature and young leaves respectively, compared to their controls (Figure 1B). Four hours after Pq exposure, ETR decreased by 24% (*p* < 0.0001) in both leaf types compared to their controls.

**Figure 2:**
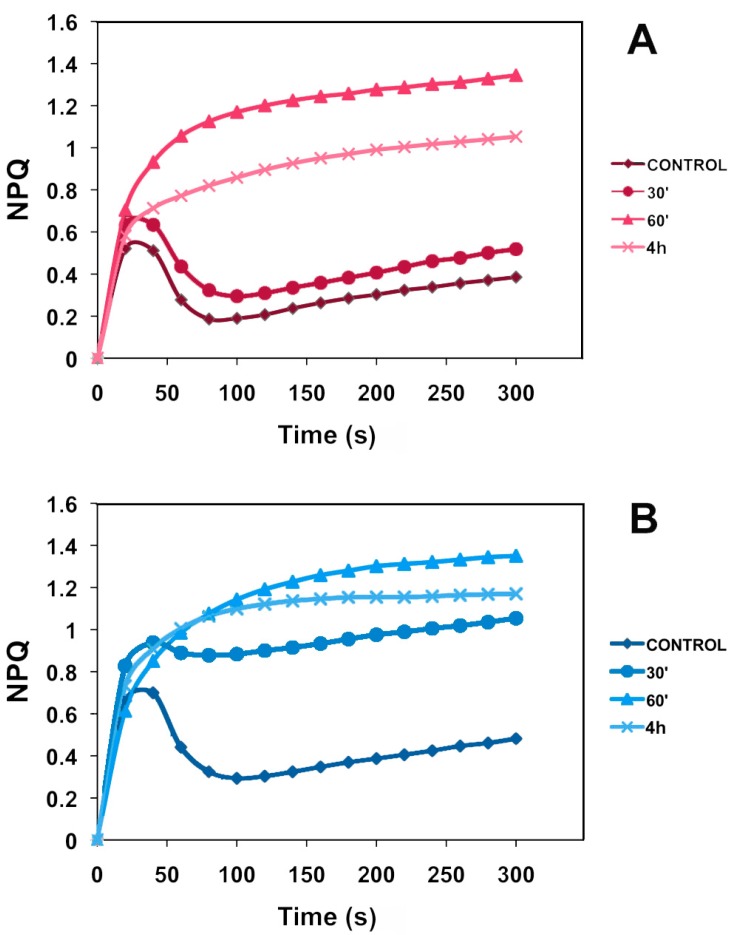
Kinetics of NPQ formation in *A. thaliana* young (**A**) and mature (**B**) leaves, during a 5-min actinic illumination with 120 μmol photons·m^−2^·s^−1^, of control (sprayed with distilled water) and paraquat (Pq) sprayed for 30 min, 60 min and 4 h. Data shown are mean values of two independent experiments each containing three leaves from three plants per treatment per experiment (*n* = 6).

The NPQ kinetics is frequently used as a tool to characterize the NPQ processes. Kinetics of NPQ formation was measured during a five-minute actinic illumination with 120 μmol photons·m^−2^·s^−1^ (Figure 2). The NPQ kinetics curves in both control leaf types and in young leaves 30 min after Pq treatment, started with a rapid initial rise (fast phase) within the first minute of illumination, followed by a decline. This fast induced and relaxing component may be attributed to qE that is fully dependent on the thylakoid lumen acidification. The NPQ kinetics curve in mature leaves 30 min after Pq treatment after the initial rise (fast phase) show a small decline before a further rise (slow phase) (Figure 2B). The NPQ kinetics curves in the 60 min and 4 h Pq-treated mature and young leaves did not show this decline after the initial rise. The highest NPQ induction values were observed 60 min after Pq treatment at both leaf types followed by 4 h and 30 min, whereas control values remained at the lowest levels.

### 2.2. Paraquat Effects on the Fraction of Open Photosystem II (PSII) Reaction Centers

**Figure 3:**
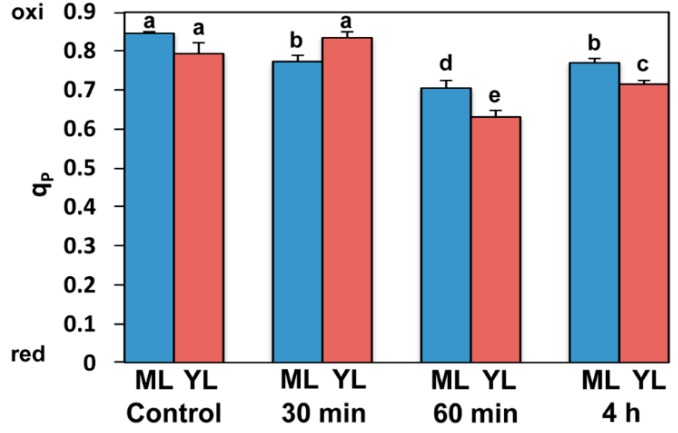
Changes of photochemical quenching (*q*_p_), the fraction of open PSII reaction centers, of control and after 30 min, 60 min, and 4 h Pq exposure of *A. thaliana* mature (ML) and young leaves (YL). Blue columns: mature leaves; red columns: young leaves. Data shown are means ± SE that is indicated by bars (*n* = 6). Columns with different lowercase letters are significantly different (*p* < 0.05).

Mature and young control leaves exhibited similar level of open PSII reaction centers, *q*_P_, which is represented by the redox state of quinone A (Q_A_) (Figure 3). Control mature leaves possessed 85% of reaction centers open while young leaves 79% of reaction centers open (Figure 3). In young leaves, 30 min after Pq treatment, this fraction of reaction centers showed a tendency to increase (ns) compared to controls, while in mature leaves it decreased by 8% (*p* < 0.05). This observation is in line with the time dependent Pq effect on the redox state of Q_A_ (Figure S1). Sixty min after Pq treatment, *q*_P_ decreased by 17% (*p* < 0.0001) and 21% (*p* < 0.0001), in mature and young leaves respectively, compared to their controls (Figure 3). Four hours after Pq treatment *q*_P_ increased by 9% (*p* < 0.05) and 13% (*p* = 0.0005) in mature and young leaves respectively, compared to 60 min, thus resulting in a decrease of 9% (*p* < 0.05) and 10% (*p* < 0.05) respectively, compared to their controls (Figure 3). Young leaves could retain a higher fraction of open PSII reaction centers than mature leaves, for almost 45 min after Pq treatment, thus a more oxidized redox state of Q_A_ (Figure S1).

### 2.3. Paraquat Effects on the Allocation of the Absorbed Light Energy in PSII

**Figure 4:**
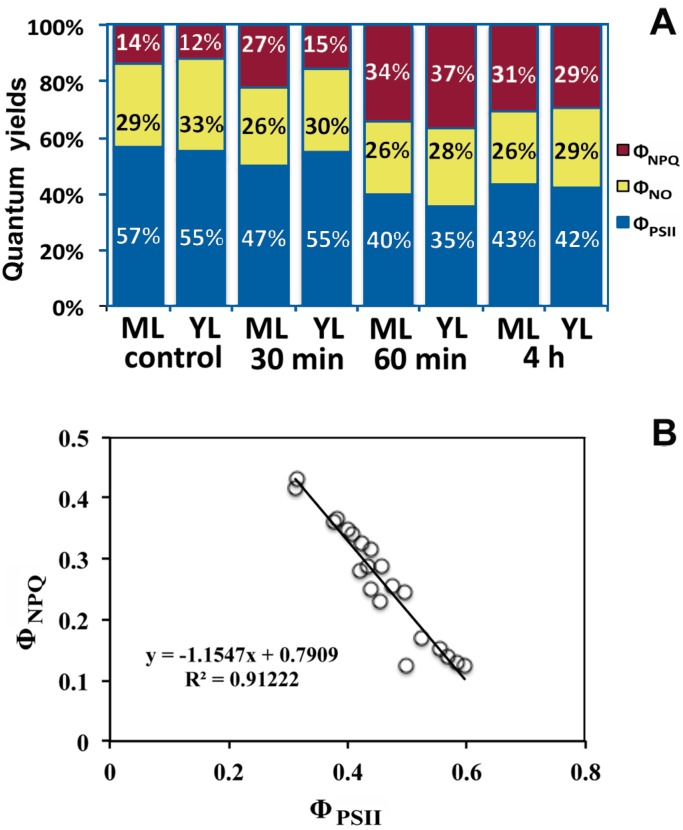
Complementary changes of (**A**) Φ_PSII_ (quantum efficiency of PSII photochemistry), Φ_NPQ_ (quantum yield for dissipation through down-regulation of PSII) and Φ_NO_ (quantum yield of non-regulated energy dissipated in PSII), revealing the allocation of the absorbed light energy in PSII, of control and 30 min, 60 min, and 4 h after Pq exposure of *A. thaliana* mature and young leaves [data shown are means (*n* = 6)]; and (**B**) relationship between Φ_PSΙΙ_ (quantum efficiency of PSII photochemistry) with Φ_NPQ_ (quantum yield for dissipation through down-regulation of PSII) of control, and after 30 min, 60 min, and 4 h Pq exposure of *A. thaliana* mature and young leaves (*R*^2^ = 0.9122, *p* < 0.0001).

Allocation of absorbed light energy to photosynthesis, dissipation and excess excitation was estimated according to the model of Kramer *et al.* [28]. This model allows assessment of excitation energy flux at PSII in three fundamentally different pathways, namely photochemical utilization, regulated heat dissipation (a loss process serving for protection), and non-regulated heat dissipation (a loss process due to PSII inactivity). These three fluxes are described by the quantum yields Φ_PSII_, Φ_NPQ_ and Φ_NO_, respectively, and add up to unity (Figure 4A). The effective quantum yield of photochemical energy conversion in PSII (Φ_PSII_), was 2% less in control young leaves compared to control mature ones (Figure 4A), indicating that a fraction of absorbed irradiance was not utilized via photochemical reactions in control young leaves and by having a 2% less energy dissipated as heat (Φ_NPQ_), possessed 4% higher non-regulated energy dissipated in PSII (Φ_NO_) than control mature leaves.

Thirty min after Pq treatment Φ_PSII_ decreased by 10% in mature leaves compared to controls, while Φ_NPQ_ increased by 13%, and these energy fluxes resulted to a decrease of Φ_NO_ by 3% compared to controls (Figure 4A). In young leaves, 30 min after Pq treatment, Φ_PSII_ remained unchanged compared to controls, Φ_NPQ_ increased by 3%, and thus Φ_NO_ decreased by 3% also (Figure 4A). Sixty min post-stress, Φ_PSII_ decreased by 17% and 20% in mature and young leaves compared to their controls, and Φ_NPQ_ increased by 20% and 25% respectively, thus resulting in a decrease of Φ_NO_ by 3% and 5% respectively (Figure 4A). With increased exposure time to Pq, the decreases of both Φ_PSII_ and Φ_NO_ resulted in an increased Φ_NPQ_, as observed in Figure 4A, since their sum is equal to unity. Four h after Pq spray, Φ_PSII_ increased by 3% and 7% in mature and young leaves respectively, compared to 60 min, indicating a recovery. However, Φ_PSII_ did not return to control levels, showing a decrease of 14% and 13% compared to control mature and young leaves respectively. This increase of Φ_PSII_, resulted in a decline of Φ_NPQ_ by 3% and 8% in mature and young leaves respectively compared to 60 min, and thus to an increase of Φ_NPQ_ by 17% in both compared to their controls (Figure 4A). A close correlation (*R*^2^ = 0.9122, *p* < 0.0001) of the decline of Φ_PSII_ under Pq treatment with the increase of Φ_NPQ_ was revealed in both leaf types (Figure 4B).

### 2.4. Ascorbate Peroxidase and Superoxide Dismutase Activity after Paraquat Exposure

**Figure 5:**
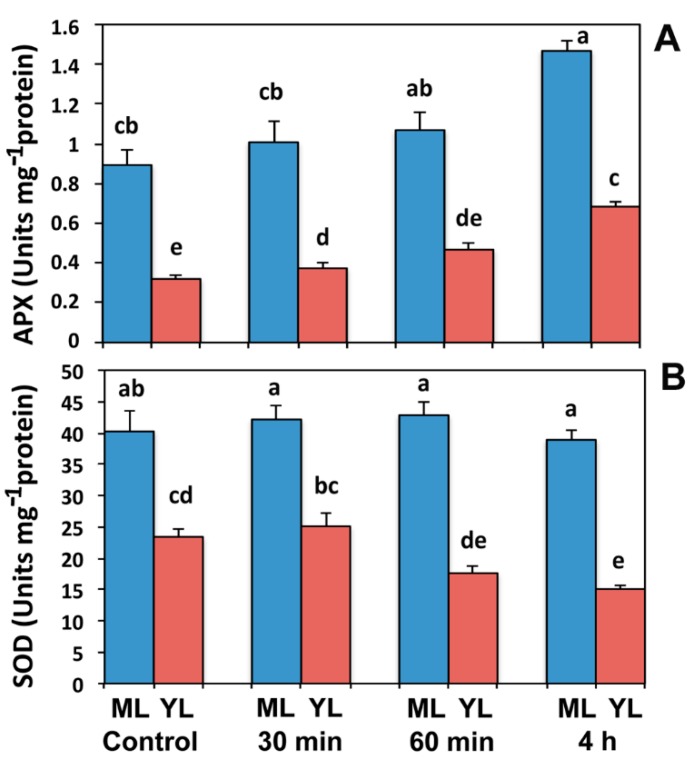
Changes of (**A**) ascorbate peroxidase activity, and (**B**) superoxidase dismutase activity, of control, and after 30 min, 60 min, and 4 h Pq exposure of *A. thaliana* mature (ML) and young leaves (YL). Blue columns: mature leaves; red columns: young leaves. Data shown are means ± SE that is indicated by bars (*n* = 6). Columns with different lowercase letters are significantly different (*p* < 0.05).

Control mature leaves in comparison to control young ones showed a higher APX activity (Figure 5A). However, in young leaves APX activity increased by 17% (*p* < 0.05), after exposure for 30 min to Pq, while in mature leaves remained unchanged. Further exposure to Pq (60 min) did not result in significant increase of APX activity in both leaf types compared to their controls. Four-hour exposure to Pq resulted in increased APX activity by 113% (*p* < 0.05) and 64% (*p* < 0.05) in young and mature leaves respectively compared to controls (Figure 5A).

Control mature leaves in comparison to control young ones showed a higher SOD activity (Figure 5B). In mature leaves SOD activity remained almost unchanged after Pq exposure, while in young leaves decreased significantly four hours after Pq exposure (36%, *p* < 0.05).

### 2.5. Imaging of Hydrogen Peroxide Production after Paraquat Exposure

**Figure 6:**
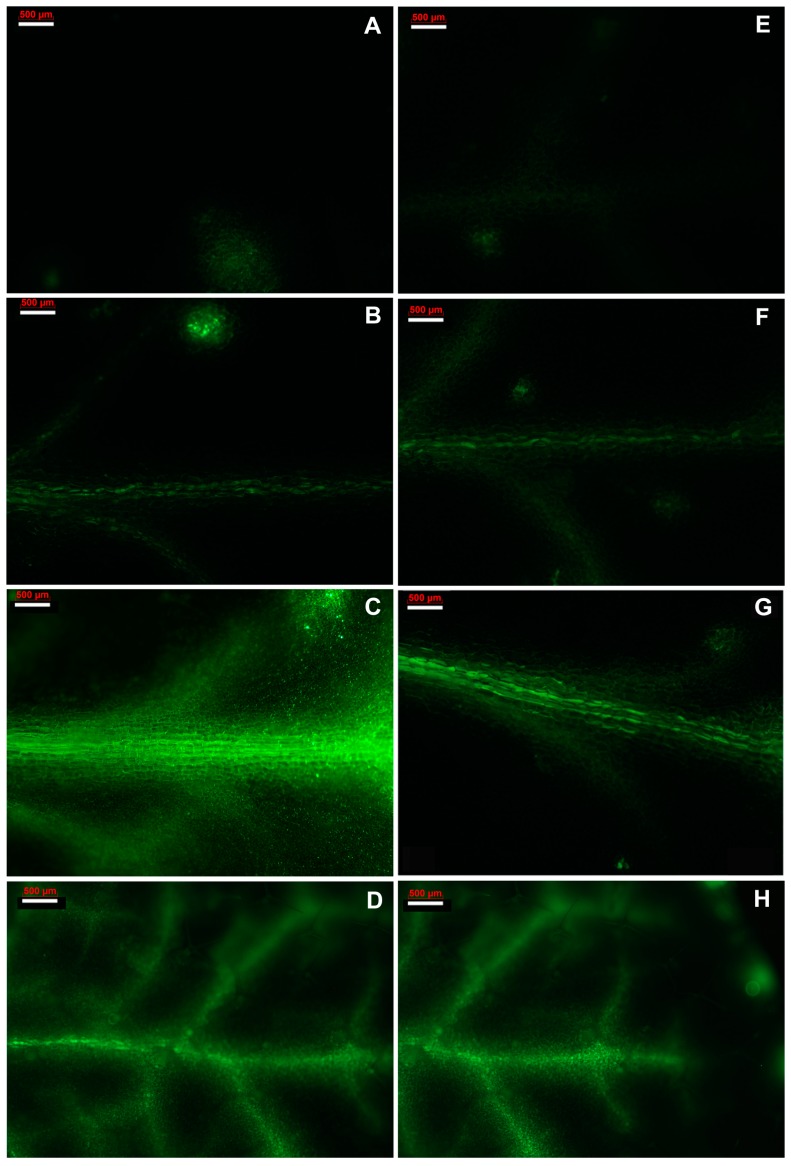
Representative patterns of H_2_O_2_ production in young (**A**–**D**) and mature (**E**–**H**) leaves of *A. thaliana* as indicated by the fluorescence of 2′,7′-dichlorodihydrofluorescein diacetate (DCF-DA), in the absence or in the presence of Pq. H_2_O_2_ content in a control young leaf (**A**) and a mature leaf (**E**); H_2_O_2_ content 30 min after Pq treatment in a young leaf (**B**) and in a mature leaf (**F**); H_2_O_2_ content 60 min after Pq treatment in a young leaf (**C**) and in a mature leaf (**G**); H_2_O_2_ content 4 h after Pq treatment in a young leaf (**D**) and in a mature leaf (**H**). Scale bars: 500 µm.

In the absence of oxidative stress no significant levels of H_2_O_2_, as indicated by the fluorescent probe 2′,7′-dichlorodihydrofluorescein diacetate (DCF-DA), could be detected in either young (Figure 6A) or mature (Figure 6E) control leaves. Exposure to Pq-induced oxidative stress resulted in production of H_2_O_2_ in both leaf types. Thirty minutes after Pq spray, the accumulation of H_2_O_2_ was almost the same in young (Figure 6B) and mature (Figure 6F) leaves. H_2_O_2_ levels increased between 30 and 60 min in both leaf types, and to a more extent in young leaves (Figure 6C), than mature (Figure 6G), reaching a maximum 60 min after initiation of Pq-induced oxidative stress. Later (4 h after Pq treatment), H_2_O_2_ levels began to drop in both leaf types (Figure 6D,H). However, H_2_O_2_ levels were still higher than those of control leaves. The increase of H_2_O_2_ production between 30 and 60 min after Pq treatment in young leaves is linked with the decrease in the fraction of open PSII reaction centers around 45 min after Pq-induced oxidative stress initiation (Figure S1).

## 3. Discussion

Tolerance to Pq-induced oxidative stress is associated with reduced photosynthetic electron flow [12] due to enhancement of NPQ that reflects the dissipation of excess excitation energy in the form of harmless heat (down regulation of PSII). This protects the plant from the damaging effects of ROS [29]. Plants have evolved multiple photoprotection mechanisms, including light avoidance through leaf and chloroplast movement, NPQ process through dissipation of absorbed light energy as thermal energy, cyclic electron transport around PSI, the photorespiratory pathway and ROS scavenging systems [19,30,31,32,33]. When these mechanisms fail to prevent damage to PSII, a complex PSII repair system is employed [32].

The “water–water cycle” [1] is mainly responsible for generation of the NPQ associated with the down-regulation of PSII [29]. In this way, protection to oxidative stress can be achieved [12]. The close correlation of the decline of Φ_PSII_ under Pq treatment with the increase of Φ_NPQ_ (Figure 4B) suggests the existence of a protective mechanism and implies a stable Φ_NO_. Indeed Φ_NO_ almost did not change during Pq treatment (Figure 4A). The non-regulated energy dissipation (Φ_NO_) consists of chlorophyll fluorescence internal conversion, and intersystem crossing which leads to the formation of singlet oxygen (^1^O_2_) via the triplet state of chlorophyll [34,35,36]. Thus, during Pq exposure the accumulation of excited species within PSII remained constant or in other words Pq exposure did not result in any increase of ^1^O_2_.

Thirty minutes after Pq exposure, young compared to mature leaves, possessed a more oxidized redox state of Q_A_ and an increased Φ_PSII_ and ETR. The slightly higher H_2_O_2_ accumulation in mature leaves compared to young leaves exposed for 30 min to Pq was due to the increase of ascorbate peroxidase (APX) activity in young leaves only (Figure 5A). However, 60 min after Pq treatment with the continued accumulation of ROS, the total antioxidant capacity of young leaves was not sufficient to scavenge the excess ROS (Figure 6C). This increase of H_2_O_2_ in young leaves 60 min after Pq treatment, contributes to the information on the redox changes in the photosynthetic electron transport chain that influences photosynthetic gene expression [37]. Over-reduction of Q_A_ indicates excess excitation energy, and thus an imbalance between energy supply and demand [38]. ROS accumulation during stress greatly depends on the balance between ROS production and scavenging [7] that in turn depends on the severity and the duration of the stress and the ability of the tissue to rapidly acclimate to the energy imbalance [39]. ROS such as the superoxide radicals and H_2_O_2_, formed at PSI are harmful byproducts in plants that cause photooxidative stress and rapidly destroy the chloroplast membranes [10,11,12] and thus they have to be scavenged by antioxidants. SOD activity was either not affected by prolonged exposure to Pq (mature leaves) or declined (young leaves) (Figure 5B), whilst APX activity increased (Figure 5A). Thus, antioxidant protection to leaf chloroplasts from ROS cytotoxicity was achieved mainly by up-regulation of APX.

The maximum Pq effect on *A. thaliana* leaves was observed 60 min post-stress, when the highest decreases of ETR and Φ_PSII_ were measured (Figure 1B and Figure 4A), and the highest increase of NPQ (Figure 1A) could not maintain a high fraction of open PSII reaction centers as that of control ones (Figure 3). This was more pronounced in young leaves that seem to suffer from oxidative stress more than mature. However even then, 63% of reaction centers remained open in young leaves (Figure 3). In accordance to this, the stable Φ_NO_ values during Pq treatment imply that photochemical conversion and photoprotective regulatory mechanisms were sufficient [23]. Maximal photoprotection can be achieved only if NPQ is regulated in such a way that PSII reaction centers remain open under given conditions [40]. In correlation to the maximum Pq effect on PSII photochemistry that was more pronounced in young leaves, H_2_O_2_ production reached a maximum 60 min post-stress and remained less in mature leaves (Figure 6C,G).

As an indicator of Pq-induced oxidative stress, H_2_O_2_ was shown to accumulate, in the leaf veins and the neighboring mesophyll cells (Figure 6), as observed also under high light stress [41]. This H_2_O_2_ accumulation in the leaf veins (Figure 6) may be a long distance signal that is induced by various abiotic stimuli [42]. H_2_O_2_ signaling has been recently reported to induce the response of favorable antioxidant and compensatory mechanisms to cope with oxidative stress [43]. Indeed, H_2_O_2_ production in young leaves 60 min after Pq treatment induced up-regulation of APX activity to provoke antioxidant protection, and thus, 4 h Pq-treated young leaves show increased APX activity compared to 60 min (Figure 5A). Oxidative damage induced by Pq in sugarcane leaves was inversely related to APX activity [44]. *A. thaliana* and tobacco plants overexpressing APX show increased resistance to Pq-induced photooxidative stress [45] and enhanced tolerance to photoinhibition at chilling temperatures [46], respectively.

Localization of H_2_O_2_ in our study contributed in elucidating the photoprotective mechanisms to Pq-induced oxidative stress conditions, confirming the conclusion by Jubany-Marí *et al.* [47] for the need of localization of H_2_O_2_ in studies for a better understanding of plant responses to stress. The time-course analysis of DCF-DA fluorescence revealed that H_2_O_2_ production reached a maximum 60 min after Pq treatment. During the maximum H_2_O_2_ production mature leaves accumulated less H_2_O_2_ than young leaves. A lower H_2_O_2_ accumulation is correlated with environmental stress tolerance [48]. Four hours after Pq treatment both leaf types seem to begin to recover as revealed by the decreased H_2_O_2_ levels (Figure 6D,H), compared to the maximum of 60 min. Furthermore, in both leaf types, ETR began to increase, as well as the fraction of open PSII reaction centers, resulting in a more oxidized redox state compared to that of 60 min Pq exposure (Figure 1B and Figure 3).

In contrast to previous reports that young leaves acclimate better to environmental stresses and to oxidative stress by a better homeostasis of ROS [18,27,49], our results do not confirm this. Thus, it seems that differences exist among the oxidative stress conditions caused by the different environmental stresses, although all of these stresses result in ROS production [50]. In accordance to this, a drought-tolerant wheat cultivar that was accompanied by a higher antioxidant system activity under drought stress was more sensitive to Pq treatment than the drought-sensitive one [51].

## 4. Experimental Section

### 4.1. Plant Material and Growth Conditions

**Figure 7:**
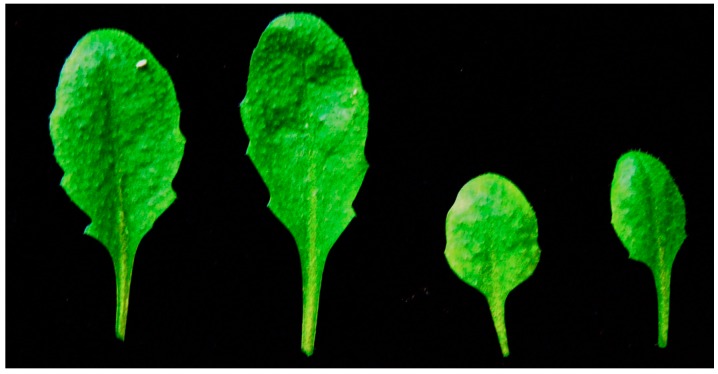
The two leaf types that were examined; fully developed mature leaves (**left**) and developing young leaves (**right**). Young leaves were considered those in the center of the leaf rosette with 1.25 ± 0.21 cm length, while mature leaves those with a length of 2.25 ± 0.25 cm.

*Arabidopsis thaliana* (L.) Heynh. (Col-0) seeds obtained from Nottingham *Arabidopsis* Stock Centre (NASC) were sown on a soil and peat mixture at (22 ± 1)/(18 ± 1) °C day/night temperature with a 16-h day at 120 ± 20 μmol photons·m^−2^·s^−1^ light intensity and (50 ± 5)%/(60 ± 5)% day/night humidity [25]. Plants were watered every two days by spraying. Four weeks after growth, the two leaf types that were examined were fully developed mature leaves and developing young leaves. The leaves in the center of the leaf rosette with 1.25 ± 0.21 cm length were considered as young expanding leaves, while mature leaves those with a length of 2.25 ± 0.25 cm (Figure 7).

### 4.2. Paraquat Treatment

Paraquat (Pq, methyl viologen, 1,1′-dimethyl 4,4′-bipyridinium dichloride) obtained from Sigma-Aldrich (Taufkirchen, Germany) was used in the experiments. After four weeks of growth, *A. thaliana* plants were sprayed with either distilled water (control) or 1 mM Pq [12]. Leaves were cut from plants 30 min, 60 min and 4 h after Pq spray and used immediately for the chlorophyll fluorescence measurements and hydrogen peroxide imaging or stored at deep freeze (−70 °C) for ascorbate peroxidase (APX) and superoxide dismutase (SOD) activity determination. Pq sprayed plants remained during treatment at the growth light intensity (120 ± 20 μmol photons·m^−2^·s^−1^).

### 4.3. Chlorophyll Fluorescence Measurements

Chlorophyll fluorescence was measured at room temperature in control (sprayed with distilled water) and after 30 min, 60 min and 4 h in Pq sprayed *A. thaliana* young and mature leaves, using an imaging-PAM fluorometer (Walz, Effeltrich, Germany), as described by Sperdouli and Moustakas [25]. All leaves were dark adapted for 15 min before measurement. Five areas of interest (AOI) were selected, one in the center of the leaf, two in the outer zone of the leaf and two in the base of the leaf. First *F*_o_ (minimum chl *a* fluorescence in the dark) and *F*_m_ (maximum chl *a* fluorescence in the dark) values were measured with dark-adapted samples. *F*_m_ was obtained with a saturating pulse of 2400 μmol photons·m^−2^·s^−1^ (800 ms) followed by application of actinic light (AL) to assess steady-state photosynthesis. A low light intensity of AL 120 μmol photons·m^−2^·s^−1^ was selected to match that of the growth light of *A. thaliana* plants, and low enough to avoid photoinhibition [27]. The illumination time was 5 min with repetitive measurements of *F*_o_′ (minimum chl *a* fluorescence in the light) and F_m_′ (maximum chl *a* fluorescence in the light) values every 20 s, from which, automatically values of other chl fluorescence parameters were calculated by the Imaging Win software. Correct *F*_o_′ determinations require the application of far red light, which could cause serious disturbances to fluorescence images. Therefore, *F*_o_′ was computed using the approximation of Oxborough and Baker [52] *F*_o_′ = *F*_o_/(*F*_v_/*F*_m_ + *F*_o_/*F*_m_). In the presence of AL, the fluorescence yield *F*_s_ (steady state chl *a* fluorescence) was measured. The calculated parameters included the effective quantum yield of photochemical energy conversion in PSII (Φ_PSII_), that estimates the efficiency at which light absorbed by PSII is used for photochemistry (*F*_m_′ − *F*_s_)/*F*_m_′. The photochemical quenching, *q*_p_, is a measure of the fraction of open PSII reaction centers that is the redox state of quinone A (Q_A_), primary acceptor of PSII, and it was calculated as (*F*_m_′ − *F*_s_)/(*F*_m_′ − *F*_o_′). The NPQ parameter, which was calculated as (*F*_m_ − *F*_m_′)/*F*_m_′, estimates the non-photochemical quenching that reflects heat dissipation of excitation energy in the antenna system. The photochemical quenching, *q*_L_, that measures the fraction of PSII centers in open state based on a lake model, was estimated as (*F*_m_′ − *F*_s_)/(*F*_m_′ − *F*_o_′) × (*F*_o_′/*F*_s_) [28]. The yield of regulated non-photochemical energy loss in PSII (Φ_NPQ_), that is the quantum yield for dissipation by down regulation in PSII, was calculated as: Φ_NPQ_ = 1 − Φ_PSII_ − Φ_NO_, and Φ_NO_, the quantum yield of non-regulated energy loss in PSII, was calculated as: Φ_NO_ = 1/[NPQ + 1 + *q*_L_ × (*F*_m_/*F*_o_ − 1)] [28]. The relative PSII electron transport rate (ETR) was calculated as: ETR = Φ_PSII_ × PPFD × *c* × abs, where *c* is 0.5 since the absorbed light energy is assumed to be equally distributed between PSII and PSI, abs is the total light absorption of the leaf taken as 0.84 and PPFD is the photosynthetic photon flux density used (120 μmol photons·m^−2^·s^−1^). NPQ kinetics were measured on *A. thaliana* young and mature leaves of control (sprayed with distilled water), 30 min, 60 min and 4 h, after Pq treatment upon illumination with AL 120 μmol photons·m^−2^·s^−1^ for 5 min. Each chlorophyll fluorescence parameter (control/treatment) represents average values from six leaf samples (each with 5 AOI) from six different plants.

### 4.4. Ascorbate Peroxidase and Superoxide Dismutase Activity

Samples of control and Pq treated *A. thaliana* leaves (100 mg) were powdered in liquid N_2_ and extracted for SOD (EC 1.15.1.1) and APX (EC 1.11.1.11) activity determination as described previously [3]. SOD activity was measured by the inhibition in the photochemical reduction of nitroblue tetrazolium (NBT) at 560 nm [53]. One unit of enzyme activity was defined as the quantity of SOD required to produce a 50% inhibition of reduction of NBT. The reaction mixture contained 50 mM Na-phosphate buffer (pH 7.8), 33 μM NBT, 10 mM l-methionine, 0.66 mM EDTA and 0.0033 mM riboflavin. APX activity was determined according to Nakano and Asada [54] following the H_2_O_2_-dependent oxidation of ascorbate at 290 nm. One unit of APX activity was defined as the amount of enzyme required for decomposing 1 μmol ascorbate·min^−1^. Protein contents were quantified at the extracts according to Bradford [55], using bovine serum albumin as a standard.

### 4.5. Hydrogen Peroxide Imaging

*A. thaliana* control and Pq sprayed young and mature leaves were incubated in darkness with 25 µM 2′,7′-dichlorofluorescein diacetate (DCF-DA) in 10 mM Tris-HCl (pH 7.4) for 30 min. After incubation, the leaves were washed three times for 10 min, in 10 mM Tris-HCl (pH 7.4), and observed under a Zeiss Axioplan Z.2 fluorescence microscope, equipped with an MRc5 Axiocam (CarlZeiss, Munich, Germany). Upon reaction with H_2_O_2_, a fluorescent DCF-derived compound is formed that can be detected by monitoring the fluorescence at excitation and emission wavelengths of 480 and 530 nm, respectively [56].

### 4.6. Statistical Analysis

A complete randomized design was utilized with six replicates and the results were expressed as means and standard error measurements. One-way ANOVA was carried out using the StatView computer package (Abacus Concepts, Inc. Berkley, Berkley, CA, USA) and means were separated at a level of *p* < 0.05.

## 5. Conclusions

The clear relationship observed between the decline of Φ_PSII_ and the increase of Φ_NPQ_, suggests the existence of an efficient protective regulatory mechanism in both leaf types under Pq treatment. Our results revealed that not only antioxidant enzyme activities but also other photoprotective mechanisms participate in *A. thaliana* leaf tolerance to Pq-induced oxidative stress.

The present study demonstrates a differential photoprotective and antioxidative mechanism of young and mature leaves to time-course Pq-induced oxidative stress. Considering leaf developmental stage is essential to the knowledge of the mechanisms underlying leaf growth responses to environmental stress [21,27,57]. Thus, in environmental stress studies, leaves of the same developmental stage should be compared [21,27,58,59]. It is concluded that tolerance to Pq-induced oxidative stress is related to the redox state of quinone A (Q_A_).

As a future perspective it would be interesting to spray a young leaf of an Arabidopsis plant and a mature one of another plant with Pq and monitor if the H_2_O_2_ production derived from the *Arabidopsis* leaves exposed to Pq, spreads throughout the vascular bundles to act as a systemic signal in the non-Pq-exposed parts of the plants. This would verify if H_2_O_2_ acts as long distance signal, a phenomenon known as “systemic acquired acclimation” [60] and would reveal a possible role of H_2_O_2_ in leaf senescence. In addition, ROS production and the differential photoprotective and antioxidant mechanisms of young *vs.* mature leaves to different Pq levels in combination to different light intensities would be interesting to study from the point of Pq application in the field under changing light intensities.

## Supplementary Materials

Supplementary materials can be found at http://www.mdpi.com/1422-0067/16/06/13989/s1.
