# Supplementary material for: Leaf Age-Dependent Photoprotective and Antioxidative Response Mechanisms to Paraquat-Induced Oxidative Stress in Arabidopsis thaliana
Source: Int J Mol Sci. 2015 Jun 18;16(6):13989–4006. doi: 10.3390/ijms160613989 (PMC4490535; doi:10.3390/ijms160613989)
Supplement: Supplementary file 1 [file ijms-16-13989-s001.pdf]

Supplementary Information

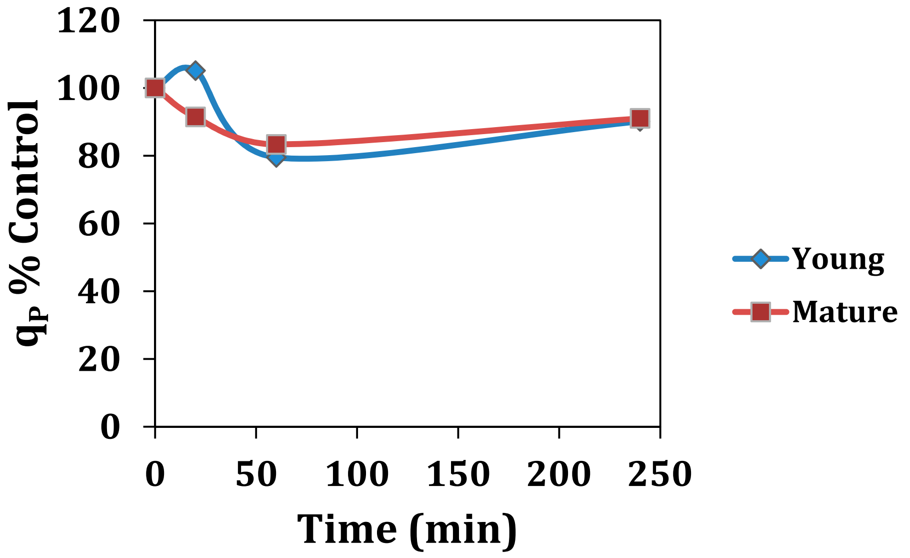

Figure S1. Time dependency of Pq effect on the redox state of quinone A (Q_A) expressed as a percentage of control young and mature leaves (sprayed with distilled water). The redox state of Q_A was recorded 30 min, 60 min, and 4 h after Pq treatment. The data were calculated from the values of the results presented in Figure 3.
